# Effectiveness of Anti-CD20 B cells depleting therapy versus conventional treatment in severe Anti-N-methyl-d-aspartate receptor encephalitis: A real-world multi-center prospective cohort study

**DOI:** 10.1016/j.neurot.2025.e00766

**Published:** 2025-10-15

**Authors:** Baojie Wang, Yuxiu Xiao, Yufeng Chu, Chunjuan Wang, Hao Sun, Teng Huang, Danqing Qin, Xuetao Cao, Shuai Guo, Haotian Zhao, Xiumin Zhao, Shougang Guo

**Affiliations:** a Department of Neurology, Shandong Provincial Hospital Affiliated to Shandong First Medical University, China; b Department of Neurology, Shandong Provincial Hospital, Shandong University, China; c Department of Neurology, Liaocheng People's Hospital, China; d Department of Neurology, Shandong Second Provincial General Hospital, China; e Department of Neurology, Jining No 1. People's Hospital, China; f Department of Neurology, Shandong Provincial Third Hospital, China

**Keywords:** B cell depleting therapy, Ofatumumab, Rituximab, Encephalitis, Prognosis

## Abstract

Approximately 25 ​%–40 ​% of anti-N-methyl-d-aspartate receptor encephalitis (NMDARE) patients develop refractory disease with prolonged neurological deficits. We aim to evaluate the efficacy of B cell depletion therapy (BCDT) via CD20 antibodies versus conventional first-line immunotherapy only (intravenous methylprednisolone for 5 days, 0.4 ​g/kg intravenous immunoglobulin for 5 days, and ≥4 consecutive plasma exchange treatments, non-BCDT group) in treatment of severe NMDARE in the real-world setting. From multicenter cohort of severe NMDARE, 108 patients (ofatumumab group 36; rituxixmab group 36; non-BCDT group 36) were prospectively reviewed. The primary end point was the proportion of patients to achieving good outcomes (modified Rankin Scale scores ≤2) at 3 months. Secondary end points included longitudinal outcomes assessed by mRS scores and Clinical Assessment Scale in Autoimmune Encephalitis (CASE) scores, cognitive function and adverse events (AEs). BCDT demonstrated a higher frequency of mRS scores ≤2 compared to non-BCDT at 3 months (ofatumumab 63.9 ​% vs. non-BCDT 36.1 ​%, p ​< ​0.001; rituximab 55.6 ​% vs. non-BCDT 36.1 ​%, p ​= ​0.007, respectively). Ofatumuamb showed superior therapeutic response at 1 month compared to both rituximab-treated patients (mean CASE score: 5.89 vs 7.91, p ​= ​0.025) and non-BCDT treated patients (mean CASE score: 5.89 vs 9.97, p ​< ​0.0001). All groups improved over 12 months, with significantly higher complete remission rates in BCDT groups (70.8 ​%–75 ​% vs. 55.6 ​%, p ​< ​0.05). Five of 33 patients (15.2 ​%) experienced persistent mild cognitive impairment. AEs were mild-to moderate in severity. We calculated the probability of relapse-free as ofatumumab and rituximab reduced risk of disease relapses compared to non-BCDT (ofatumumab vs. non-BCDT: HR, 0.193; 95 ​% CI, 0.062–0.6; p ​= ​0.007; rituximab vs. non-BCDT: HR, 0.265; 95 ​% CI, 0.089–0.787; p ​= ​0.029). Therefore, we suggest that BCDT effectively promoted neurological recovery, and reduced relapse risk with favorable safety, while ofatumumab demonstrated superior efficacy in controlling early disease severity.

## Introduction

Anti-N-methyl-d-aspartate receptor encephalitis (NMDARE) generally manifests a spectrum of neuro-psychiatric manifestations including abnormal psychiatric/behaviour or cognitive dysfunction, speech dysfunction, seizures, movement disorder, decreased level of consciousness, autonomic dysfunction and central hypoventilation [1]. First-line immunotherapy remains an important therapeutic option for patients with NMDARE [2,3]; however, meaningful clinical response display heterogeneous, with approximately 25 ​%–50 ​% of patients being refractory to first-line immunotherapy and have severely prolonged neurological deficits [[4], [5], [6], [7]].

B cells are known to contribute to the pathogenesis of NMDARE by driving the production of disease specific autoantibodies, secreting proinflammatory cytokines [8]. The important role of B cells is also supported by the high efficacy and relative safe of specific CD20 B cell depleting drug, which have been proven by findings from case series as well as observational studies [[9], [10], [11], [12], [13], [14], [15]]. Rituximab (RTX) is a chimeric IgG1 monoclonal antibody that targets CD20 on B cells, resulting in the depletion of pathogenic B cells. Preliminary evidence was found of improved effectiveness compared with first-line therapy alone [11]. Over time, new anti-CD20 monoclonal antibodies, such as ofatumumab (OFA), a fully human antibody that first used as disease-modifying therapies for multiple sclerosis [16], recognizes an epitope encompassing the small and large extracellular loops of CD20 receptors and has more efficient complement-dependent cytotoxicity in vitro [17]. Regarding autoimmune encephalitis (AE), successful treatment by OFA were reported [10,14,15].

Both highly targeted biologic therapies, such as OFA and RTX, may be considered as treatment options for severe NMDARE. However, rarity of NMDARE limits the ability to conduct randomized control trials (RCTs). To date, directly comparative studies for the efficacy and safety of OFA, RTX and non-BCDT for severe AE are lacking. We therefore present data to advance knowledge and clinical care in severe NMDARE in this prospective, real-wold multicentric study utilizing a cohort of NMDARE patients treated at five neuroimmunological centers.

## Methods

### Standard protocol approvals and patient consents

The study was conducted according to the Declaration of Helsinki and approved by the Ethics Committees of the Shandong Provincial Hospital. Patients were enrolled from January 2018 to December 2024, informed consents were signed by the participants or the legal representatives.

### Study design and population

Our cohort is a prospective, multicenter observational study, recruited patients from five hospitals, all are certified specialized ward for neuroimmunological diseases, employing standardized workflows for patient management. At each participating center, a uniform Case Report Form (CRF) was used to document medication administration time point and frequency, recording adverse events, concomitant medications and relapse. A total of 235 patients with NMDARE were recruited. Diagnosis of severe NMDARE was established by characteristic clinical presentation and supported by NMDAR-IgG findings in accordance with the following criteria: 1) patients older than 14 years of age; 2) met the criteria for a clinical diagnosis of AE [18]; 3) the presence of cerebrospinal fluid (CSF) autoantibodies to NMDAR by cell-based assays (CBAs); 4) severe neurological dysfunction at baseline visit with a modified Rankin scale (mRS) score of 4–5; Additionally, the following exclusion criteria was applied during patient selection: 1) patients with concurrent systemic autoimmune disease; 2) positive serum and/or CSF laboratory tests for other neuronal surface or intracellular antigens; 3) pregnancy during the treatment period; 4) presence of a biological agent other than OFA or RTX at disease onset; 5) malignant tumor with ongoing chemotherapy; 6) patients with active Hepatitis B virus or human immunodeficiency virus.

Following selection using the inclusion and exclusion criteria, 108 patients were included in the analysis.

### Treatment protocols

For eligible patients, treatment allocation followed a real-world clinical decision-making framework in accordance with clinical severity, preferences of their legal representatives, drug availability/route of administration, and the anticipated risk of infusion reactions. Three treatment strategies were defined: 1) the OFA group (initiating OFA therapy); 2) the RTX group (undergoing RTX therapy); and 3) the non-BCDT control group (receiving only conventional first-line immunotherapy without subsequent BCDT).

B cell depleting therapy were administrated after at least 10–14 days from first-line treatment initiation when patients showed an improvement in the mRS score <1 point. OFA was administrated with a dosage of 20 ​mg subcutaneously every 4 weeks after 20 ​mg loading doses at days 1, 7, and 14. The RTX dosing regimen was based on previous studies in AE, demonstrating that efficacy could be achieved with reduced doses to minimize toxicity [11,13,19]. Patients received either 100 ​mg weekly for four weeks [13] or a single dose of 375 ​mg/m^2^ [19], followed by a repeat doses of 100 ​mg when the proportion of circulating CD19^+^ B cells exceeded 1 ​% of peripheral blood mononuclear cells (PBMCs).

### Analysis of laboratory and clinical profiles

Baseline data included sex, age at disease onset. Symptom profiles were analyzed according to the following categories: abnormal psychiatric behavior or cognitive dysfunction, speech dysfunction (pressured speech, verbal reduction, mutism), seizures, movement disorder, dyskinesias, rigidity or abnormal postures, decreased level of consciousness, autonomic dysfunction, or central hypoventilation.

Laboratory data, including brain magnetic resonance imaging (MRI), cerebrospinal fluid (CSF), and electroencephalography (EEG) scans obtained before the first-line treatment were reviewed. In the MRI analysis, we investigated the presence of increased T2 or fluid-attenuated inversion recovery parenchymal signal intensity or contrast enhancement on brain MRI. CSF analysis included the presence of CSF lymphocytosis (>10 count/mm^3^), or protein level elevation (>0.45 ​mg/dL). The EEG analysis investigated the presence of epileptic discharges. To screen for an associated neoplasm, all patients underwent a CT scan of the thorax/abdomen/pelvis and ultrasound of the abdomen and the pelvic region.

B cell levels in peripheral blood were measured by flow cytometry using fluorescence-activated cell sorting (FACS), conducted at laboratory in the 24 ​h after sample collection. Immunoglobulin (IgG, IgA, IgM) were detected by using a nephelometer. In addition, levels were compared with normal values (normal ranges: IgG: 7.2–15.6 ​g/L; IgA: 0.67–3.14 ​g/L; IgM: 0.5–2.55 ​g/L).

AE-related clinical scores were prospectively assessed at baseline and at follow-up using the mRS scores and Clinical Assessment Scale in Autoimmune Encephalitis (CASE) scores for disease severity by the treating neurologist [5]. The anti-NMDAR Encephalitis One-Year Functional Status (NEOS, range from 0 to 5) score was assessed at baseline [4]. Neurocognitive performance was assessed using the Mini-Mental State Examination (MMSE). Patient-reported outcome for measuring health status or medical outcomes were 36-item Short Form Health Survey (SF-36) scores including the domains of physical functioning (PF), role-physical (RP), bodily pain (BP), general health (GH), vitality (VT), social functioning (SF), role-emotional (RE), mental health (MH), health transition (HT), physical and mental health summary measures (PCS&MCS) [20,21]. We simultaneously matched a healthy control group identical in age and sex for comparative analysis.

Assessments were performed at baseline, monthly during the first 6 months, every 2 months until month 12, and every 3 months thereafter. At each study visit, we performed careful clinical examinations, recorded the clinical score, concomitant medications given and adverse events.

### Outcomes, adverse effects

The primary end point was the proportion of patients to achieve good outcome (mRS ​= ​0–2) at 3 months. Secondary end point were as follows: 1) 1 month initial clinical efficacy; 2) the sustained longitudinal outline of clinical outcomes (change in scores of mRS, CASE, MMSE at 1, 3, 6, 9, 12 months and final follow-up); 3) SF-36 score at final follow-up; (4) the median time to reach good outcome; 5) the number of patients with relapse and the difference in the time to relapse; 6) the dosage of corticosteroid at 6 months.

Adverse events were recorded at all clinical visits and graded according to the Common Toxicity Criteria version 5.0. Patients who experienced severe adverse effects would exit the study, as determined by physicians [22].

### Definitions

Non-BCDT was defined as patients who received first-line treatment only. First-line treatment was defined as 500–1000 ​mg intravenous methylprednisolone (IVMP) for 5 days, 0.4 ​g/kg intravenous immunoglobulin (IVIG) for 5 days, and ≥4 consecutive plasma exchange treatments. Baseline visit were assessed by neurologists after 10–14 days from the first-line treatment initiation. Across BCDT groups, add-on immunotherapy (daratumumab or bortezomib) were used as rescue therapy if the disease worsened or showed no improvement after BCDT. Maintenance immunotherapy (6 months or more) included daily oral corticosteroids, RTX or OFA redosing. The depletion of CD19^+^ B cells was defined as below 1 ​% of PBMC. Relapse was defined as new onset or worsening of symptoms occurring after initial improvement or stabilisation of at least 2 months [2]. MMSE scores were categorized as follows: ≥27 (normal), 21–26 (mild impairment), 10–20 (moderate impairment), and ≤9 (severe impairment). Good outcome was defined as mRS ​= ​0–2, unfavorable outcome as mRS ​= ​3–6, complete remission as mRS ​= ​0.

### Statistical analysis

Continuous data are presented as mean (SD), median and interquartile range (IQR), categorical variables are presented as absolute frequencies and percentages. Differences in baseline characteristics between groups were evaluated with a 2-tailed Fisher exact test or Pearson's χ2 test for categorical variables and Kruskal-Wallis test with Dunn's procedure for continuous variables. Disease-related clinical scores changes over time relative to baseline are displayed as mean along with 95 ​% confidence intervals (CIs) and were analyzed using analysis of variance (ANOVA), followed by Tukey test for normally distributed variables. Odds ratios (ORs) and 95 ​% CI for the associations between functional outcome at 3 months and potential covariates were estimated by logistic regressions model. Group differences in time-to-event outcome (duration from baseline to relapse) were assessed through Kaplan-Meier curves, with statistical significance assessed using the log-rank test. Data were analyzed using SPSS (version 26) and GraphPad Prism software (8.4.2). Statistical significance was defined as two-sided p values ​< ​0.05.

### Data availability

Anonymized data not published within this article will be made available by request from any qualified investigator.

## Results

### Characteristics of patients and immunotherapy

**Fig. 1:**
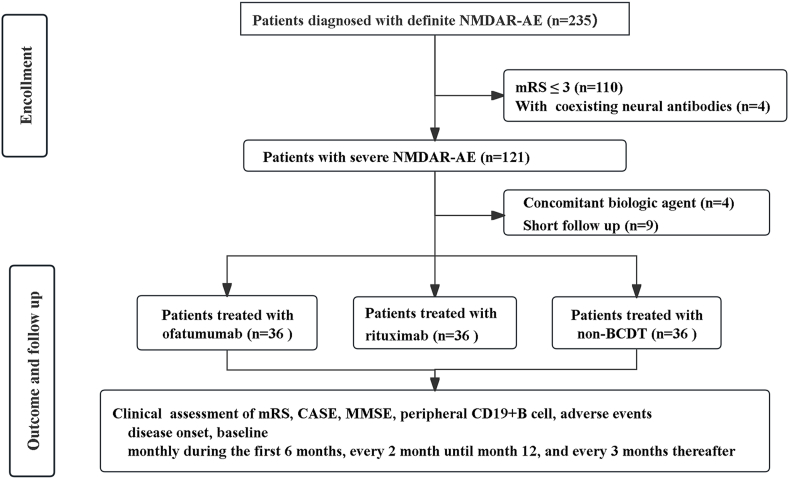
Flowchart of study population.

All patients were prospectively included between January 2018 and December 2024. We identified 235 patients with anti-NMDAR encephalitis of whom 108 were enrolled in the final analysis and were followed-up for 24 (15–36) months. A total of 36 patients starting with OFA, 36 patients starting with RTX and 36 patients treated without BCDT (Fig. 1).

The median age at disease onset was 29 (18–38) years, with 31 of 108 patients (28.7 ​%) 18 years or younger at onset. A total of 6 of 59 patients (10.2 ​%) had a ovarian teratoma. The most frequent AE symptoms were disturbance of consciousness (n ​= ​78, 72.2 ​%), seizures or status epilepticus (n ​= ​78, 72.2 ​%), cognitive impairment (n ​= ​54, 50 ​%) and neuropsychiatric symptoms (n ​= ​52, 48.1 ​%). 76 of 108 patients (70.4 ​%) needed ICU admission with a median ICU stay of 29 (26–37) days due to respiratory failure requiring ventilator support, status epilepticus or decreased consciousness. Clinical severity as assessed by mRS scores or CASE scores and corticosteroid dose at baseline were balanced between all groups.

First-line immunotherapy was applied in all patients with IVMP (n ​= ​108, 100 ​%), IVIG (n ​= ​100, 92.6 ​%), plasma exchange (n ​= ​13, 12 ​%). The duration between the end of first-line therapy and begin of BCDT were 15 (13–27) days in OFA group and 14 (10–16) days in RTX group.

Baseline characteristics, treatment profiles are detailed in Table 1 and eTables 1-2.

**Table 1:** Baseline demographic and clinical characteristics.

Characteristics	Total (n ​= ​108)	Ofatumumab (n ​= ​36)	Rituxumab (n ​= ​36)	Non-BCDT (n ​= ​36)	P value
Age, year, median (IQR)	26 (18–36)	22 (19–44)	22 (16–37)	29 (23–35)	0.566
Female, n (%)	59 (54.6)	17 (47.2)	22 (61.1)	20 (55.6)	0.131
Symptoms, n (%)					
Seizures	78 (72.2)	28 (77.8)	26 (72.2)	24 (66.7)	0.219
Psychosis	52 (48.1)	15 (41.7)	20 (55.6)	17 (47.2)	0.133
Cognitive disorder	54 (50)	16 (44.4)	18 (50)	20 (55.6)	0.237
Disturbance of consciousness	78 (72.2)	28 (77.8)	26 (72.2)	24 (66.7)	0.219
Speech disturbance	38 (35.2)	11 (30.6)	14 (38.9)	13 (36.1)	0.489
Dyskinesias and movement disorders	41 (38)	14 (38.9)	12 (33.33)	15 (41.7)	0.41
Central hypoventilation	9 (8.3)	4 (11.1)	3 (8.3)	2 (5.5)	0.294
ICU admission, n (%)	76 (70.4)	25 (69.4)	27 (75)	24 (66.7)	0.436
ICU stay, days, median (IQR)	29 (26–37)	35 (27–39)	28 (24–38)	30 (28–36)	0.565
Length of hospital stay, days, median (IQR)	37 (27–45)	40 (30–46)	39 (30–55)	37 (27–43)	0.36
Teratoma, n (%)	6 (10.2)	1 (2.7)	2 (5.5)	3 (8.8)	0.203
Auxiliary examination, n (%)					
MRI abnormalities	54 (50)	16 (44.4)	20 (55.6)	18 (50)	0.237
EEG abnormalities	54 (50)	18 (50)	20 (55.6)	16 (44.4)	0.119
CSF abnormalities	55 (50.9)	18 (50)	17 (47.2)	20 (55.6)	0.237
CD19^+^B cell count, %, median (IQR)	20.82 (15.7–28.08)	18.69 (15.9–28.07)	22.16 (15.38–27.27)	22.7 (15.5–33.72)	0.604
NMDAR antibody positive, n (%)					
Serum	73 (67.6)	25 (69.4)	22 (61.1)	26 (72.2)	0.229
CSF	104 (96.3)	34 (94.4)	35 (97.2)	35 (97.2)	0.458
NEOS scores	2 (1–3)	2 (1–3)	2 (1–2)	2 (1–3)	0.183
First-line treatment, n (%)					
IVMP	108 (100)	36 (100)	36 (100)	36 (100)	>0.99
IVMP ​+ ​IVIG	100 (92.6)	32 (88.9)	33 (91.6)	35 (97.2)	0.09
IVMP ​+ ​IVIG ​+ ​PLEX	11 (10.2)	2 (5.6)	1 (2.7)	8 (22.2)	<0.001
mRS score at baseline, median (IQR)	4 (4–5)	4 (4–5)	4 (4–5)	4 (4–4)	0.35
CASE score at baseline, median (IQR)	15 (13–19)	17 (12–21)	16 (13–19)	15 (13–18)	0.576
Cortisone at baseline, mg, mean (SD)	120.7 (61.38)	107.2 (42.53)	125.6 (68.89)	129.4 (68.37)	0.263
Time from the end of first-line treatment to BCDT, days, median (IQR)	14 (10–20)	15 (13–27)	14 (10–16)	/	/

**Abbreviations:** BCDT, B cell depleting therapy; ICU, intensive care unit; MRI, magnetic resonance imaging; CSF, cerebrospinal fluid; EEG, electroencephalogram; NMDAR, N-methyl-d-aspartate receptor; NEOS, the NMDAR Encephalitis One-Year Functional Status; IVIG, IV immunoglobulin; IVMP, intravenous methylprednisolone; PLEX, plasma exchange; mRS, modified Rankin scale; CASE, Clinical Assessment Scale in Autoimmune Encephalitis; IQR, interquartile range.

### Clinical outcomes and response to immunotherapy

Table 2 shows the response and clinical outcomes in all groups. The initial clinical response was assessed at the end of the first month from baseline. Patients treated with BCDT were more likely to achieve ≥2 point mRS score improvement (OFA: n ​= ​13, 36.1 ​%; RTX: n ​= ​12, 33.3 ​%; non-BCDT treatment: n ​= ​4, 11.1 ​%; p ​< ​0.001).

**Table 2:** Response and clinical outcomes.

Outcomes	Total (n ​= ​108)	Ofatumumab (n ​= ​36)	Rituxumab (n ​= ​36)	Non-BCDT (n ​= ​36)	P value		Post hoc	
**Initial response at 1 month from baseline time point**								
△mRS score≥1 point, n (%)	93 (86.1)	33 (91.7)	31 (86.1)	29 (80.6)	0.077		**0.037**^c^	
△mRS score≥2 point, n (%)	29 (26.9)	13 (36.1)	12 (33.3)	4 (11.1)	**<0.001**		**<0.001**^c^	**0.001**^d^
△CASE score, mean (SD)	7.91 (3.42)	10.2 (3.71)	8.31 (2.49)	5.2 (1.64)	**<0.001**	**0.014**^b^	**<0.001**^c^	**0.001**^d^
**Sustained response from baseline time point**								
Favorable outcome (mRS score ≤2), n (%)								
3 months	56 (51.9)	23 (63.9)	20 (55.6)	13 (36.1)	**<0.001**		**<0.001**^c^	**0.007**^d^
6 months	74 (68.5)	28 (77.8)	26 (72.2)	20 (55.6)	**0.003**		**0.002**^c^	**0.027**^d^
9 months	88 (81.5)	32 (88.9)	30 (83.3)	26 (72.2)	**0.008**		**0.004**^c^	
12 months	99 (91.7)	35 (97.2)	34 (94.4)	30 (83.3)	**0.001**		**0.002**^c^	**0.025**^d^
last follow-up	103 (95.4)	35 (97.2)	34 (94.4)	34 (94.4)	0.532			
Complete remission at 12 months, n%	64 (66.7)	17 (70.8)^a^	27 (75)	20 (55.6)	**0.009**		**0.027**^c^	**0.007**^d^
Complete remission at at follow-up, n%	90 (83.4)	30 (83.3)	32 (88.9)	28 (77.8)	0.113			
Median time to achieve mRS≤2 from baseline time point, days, median (IQR)	85 (65–170)	78 (50–135)	83 (34–152)	145 (80–270)	**0.012**		**0.014**^c^	**0.049**^d^
Cortisone dosage at 6 months, mg, mean (SD)	14.21 (8.79)	10.79 (7.04)	12.94 (8.08)	18.86 (9.24)	**<0.001**		**<0.001**^c^	**0.009**^d^
Cortisone withdraw at 6 months, n (%)	23 (22.1)	13 (37.1)	7 (20.6)	3 (8.6)	**<0.001**	**0.019**^b^	**<0.001**^c^	**0.028**
Maintenance immunotherapy, n (%)	85 (81.7)	27 (75)	28 (77.8)	30 (83.3)	0.36			
Relapse during follow-up, n (%)	16 (14.8)	2 (5.6)	4 (11.1)	10 (27.8)	**<0.001**		**<0.001**^c^	**0.008**^d^
Timing of relapse, months, median (IQR)	11 (8–14)	12 (16–20)	16 (11–27)	9 (6–11)	**0.019**			
Follow up duration, months, median (IQR)	24 (15–36)	13 (8–19)	28 (19–38)	30 (24–36)	**<0.001**	**<0.001**^b^	**<0.001**^c^	
Death, n (%)	4 (3.7)	1 (2.7)	2 (5.6)	1 (2.7)	0.458			
Adverse evens, n (%)								
Infusion/injections-related reactions								
Influenza-like symptoms	13 (12)	6 (16.7)	4 (11.1)	3 (8.3)	0.137			
Arrhythmia	7 (6.5)	1 (2.7)	5 (13.9)	1 (2.7)	**0.002**	**0.009**^b^		**0.009**^d^
Infection	15 (13.9)	5 (13.9)	7 (19.4)	3 (8.3)	0.077			**0.037**^d^
Hypogammaglobulinemia	22 (30.6)	10 (27.8)	12 (33.3)	/	/			
Depletion of CD19^+^ B cells after first dose, n%	62 (86.1)	30 (83.3)	32 (88.9)	/	/			
Total cumulative dosage of BCDT, mg; median (IQR)	/	120 (80–200)	500 (400–600)	/	/			

Abbreviations: △mRS, the change in mRS scores; IQR, interquartile range; SD, Standard Deviation; mRS, modified Rankin scale; BCDT, B cell depleting therapy.

a For 36 patients treated with ofatumumab group, the follow-up period for 24 patients exceeded 1 year.

b p ​< ​0.05 for ofatumumab group vs. rituximab group.

c p ​< ​0.05 for ofatumumab group vs. non-BCDT group.

d p ​< ​0.05 for rituximab group vs. non-BCDT group.

**Fig. 2:**
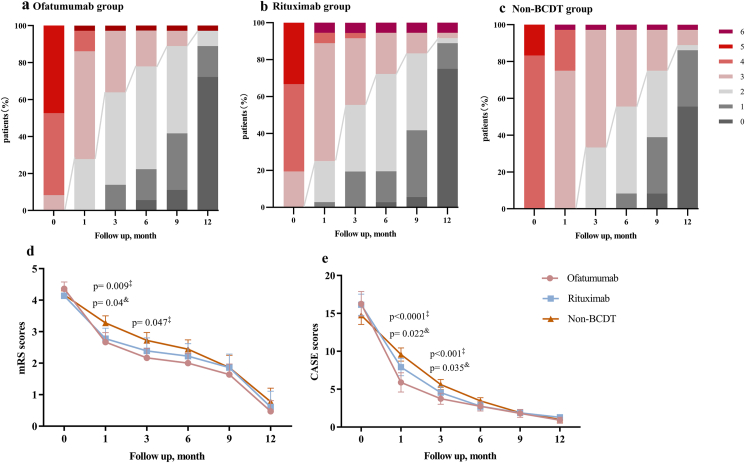
**Clinical Outcomes (A–C)** Functional outcomes during the first 12-month follow-up for ofatumumab group(A), rituximab group(B) and non-BCDT group(C). The line represents the change in mRS scores dividing favorable mRS scores (0–2) and unfavorable mRS scores (≥3); (D–E) Longitudinal profile of mRS score(D) and CASE score(E) improvement during the first 12-month follow-up among three group ‡p ​< ​0.05 for ofatumumab group vs. non-BCDT group; &p ​< ​0.05 for rituximab group vs. non-BCDT group; Error bars indicate the 95 ​% confidence intervals.

At 3 months, OFA group and RTX group had more frequent mRS scores≤2 as compared with non-BCDT group (OFA 63.9 ​% vs. non-BCDT 36.1 ​%, p ​< ​0.001; RTX 55.6 ​% vs. non-BCDT 36.1 ​%, p ​= ​0.007, respectively) (Fig. 2A-**C)**. Patients receiving BCDT demonstrate a significantly lower scores on the mRS and CASE than those treated with non-BCDT group at 1 month and 3 months (Fig. 2D and E). Additionally, OFA-treated patients demonstrated a significantly greater benefit from treatment as compared with RTX patients (mean [SD] CASE score: 5.89 [3.68] vs. 7.91 [3.34], p ​= ​0.025) and non-BCDT treated patients (mean [SD] CASE score: 5.89 [3.68] vs. 9.97 [2.42], p ​< ​0.0001) at 1 month from baseline.

**Fig. 3:**
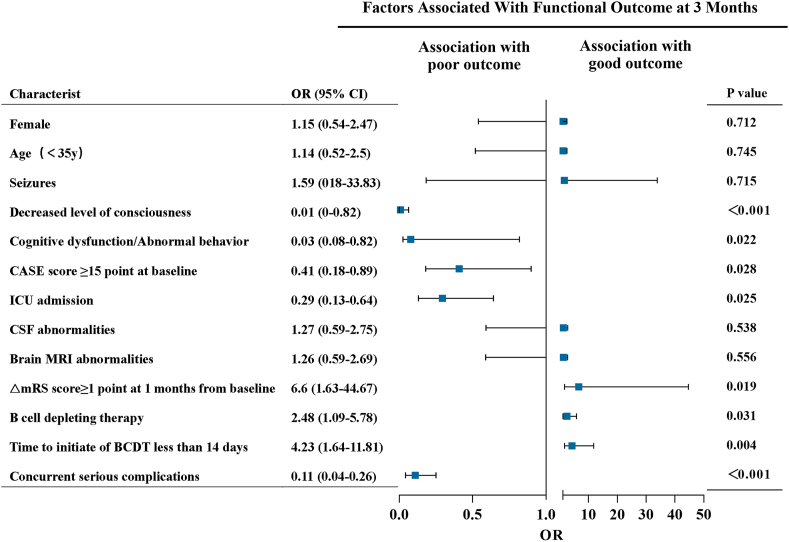
**Factors associated with functional outcome at 3 months** OR, Odds ratios (ORs); CIs, confidence intervals; △mRS, the change in mRS scores; ICU, intensive care unit; MRI, magnetic resonance imaging; CSF, cerebrospinal fluid; modified Rankin scale; CASE, Clinical Assessment Scale in Autoimmune Encephalitis; BCDT, B cell depleting therapy.

We next assessed factors associated with functional outcome at 3 months. In the logistic regression model, factors significantly related to poor outcome were decreased level of consciousness (OR, 0.01; 95 ​% CI, 0–0.82; p ​< ​0.001), cognitive dysfunction or abnormal behavior (OR, 0.03; 95 ​% CI, 0.08–0.82; p ​= ​0.022), CASE score ≥15 points at baseline (OR, 0.41; 95 ​% CI, 0.18–0.89; p ​= ​0.028), ICU admission (OR, 0.29; 95 ​% CI, 0.13–0.64; p ​= ​0.025) and concurrent serious complications (OR, 0.11; 95 ​% CI, 0.04–0.26; p ​< ​0.001). Treatment factors associated with increased odds of good outcome were use of BCDT (OR, 2.48; 95 ​% CI, 1.09–5.78; p ​= ​0.031), time to initiate of BCDT less than 14 days (OR, 4.23; 95 ​% CI, 1.64–11.81; p ​= ​0.004) and △mRS score≥1 point at 1 month from baseline (OR, 6.6; 95 ​% CI, 1.63–44.67; p ​= ​0.019) (Fig. 3). During the follow-up period, the median time to achieve good outcome was 78 (50–135) days in the OFA group compared with 83 (34–152) days in the RTX group (p ​> ​0.99) and 145 (80–270) days in non-BCDT group (p ​= ​0.014).

Sustained and long-term improvements were observed in mRS scores and CASE scores in all groups (eTable 3-5). Least squares mean (95 ​% CI) change in mRS scores at 6 months were −2.36(−1.75, −2.97; OFA), −2.14 (−1.27, −3.01; RTX) and −1.72 (−1.18, −2.27; non-BCDT) respectively. By 12 months from the baseline time points, OFA group (n ​= ​17, 70.8 ​%) and RTX group (n ​= ​27, 75 ​%) showed a significantly higher frequency of complete remission compared with the non-BCDT group (n ​= ​20, 55.6 ​%) (p ​= ​0.027 and p ​= ​0.007, respectively). At the last follow-up, we detected no significant difference between all groups regarding the proportion of good outcome and complete remission. The use of rescue therapies was significantly lower in patients treated with OFA (n ​= ​1, 2.7 ​%) as compared with RTX group (n ​= ​5, 13.9 ​%, p ​= ​0.009).

### Maintenance immunotherapy

**Fig. 4:**
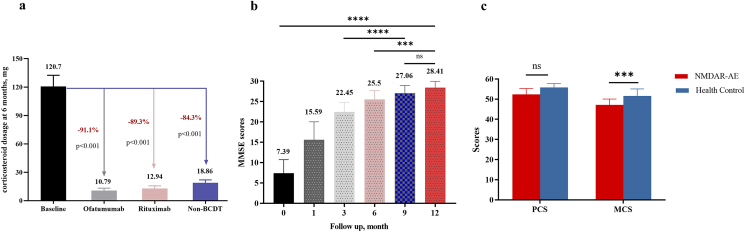
**Secondary Outcome of Corticosteroid Use, Longitudinal Cognitive and Health-Related Quality of Life** (A) Mean change over time in disease outcome in corticosteroid use; (B) Longitudinal Assessment of Cognitive Function. MMSE scores over time in patients with NMDARE who presented with cognitive deficits at baseline. The assessment was conducted at baseline (month 0), 1, 3, 6, 9, and 12 months. A higher score indicates better cognitive performance; (C) SF-36 score in patients with NMDARE and health control. Datas are presented as mean with error bars representing the 95 ​% confidence intervals. MMSE, Mini-Mental State Examination; BCDT, B cell depleting therapy; MCS, Mental Component Summary; PCS, Physical Component Summary; ns, not significant; NMDARE, N-methyl-d-aspartate receptor autoimmune encephalitis; ∗∗, p ​< ​0.01; ∗∗∗, p ​< ​0.001; ∗∗∗∗, p ​< ​0.0001.

Eighty-five patients (81.7 ​%) received maintenance immunotherapy. Among patients taking corticosteroid at 6 months from baseline time points, all groups were able to reduce corticosteroid doses. The mean (SD) daily dose decreased from 120.7 (61.38) mg/day to 14.21 (8.79) mg/day, a decrease of approximately 87.6 ​% (p ​< ​0.0001) (Fig. 4A). Patients receiving BCDT taking lower dose of corticosteroid compared with non-BCDT treatment (mean [SD] dosage: OFA 10.79 [7.04] mg/day, RTX 12.94 [8.08] mg/day, non-BCDT 18.86 [9.24] mg/day; p ​< ​0.001). A total of 23 of 104 patients (22.1 ​%) had completely discontinued corticosteroid at 6 months (OFA: n ​= ​13; RTX: n ​= ​7; non-BCDT: n ​= ​3; p ​< ​0.001). Forty-four patients were reinfused with RTX (n ​= ​20) and OFA (n ​= ​24) for 6 months or more.

### Longitudinal cognitive function and health-related quality of life

Cognitive testing was performed in 45/54 (83.3 ​%) patients with cognitive deficit. Among 33 patients who were assessed prospectively for several time in MMSE score, 26 of 33 patients (78.8 ​%) had sustained improvement lasting to 9 months from baseline (Fig. 4B). At 12 months, the least squares mean (95 ​% CI) change in MMSE scores was −21.02 (−23.11, −18.93) (p ​< ​0.0001),significantly improved from baseline. After a median follow-up of 24 (15–36) months, 5 of 33 patients (15.2 ​%) experienced persistent mild cognitive impairment. Sequelae remained in the patient-reported outcomes. The Mental Component Summary (MCS) score for patients with NMDARE (median 51, IQR 39–56) was significantly lower than that of the healthy control group (median 58, IQR 43–61; p ​< ​0.001) (Fig. 4C). Additionally, NMDARE patients had significantly lower scores in the role-emotional (RE; p ​= ​0.045) and social functioning (SF; p ​< ​0.001) domains. The Physical Component Summary (PCS) score did not differ significantly between groups. However, scores for three PCS component domains—role-physical (RP; p ​< ​0.001), physical functioning (PF; p ​= ​0.013), and general health (GH; p ​= ​0.001), were significantly lower in patients than in controls (eTable 6).

### Relapses

**Fig. 5:**
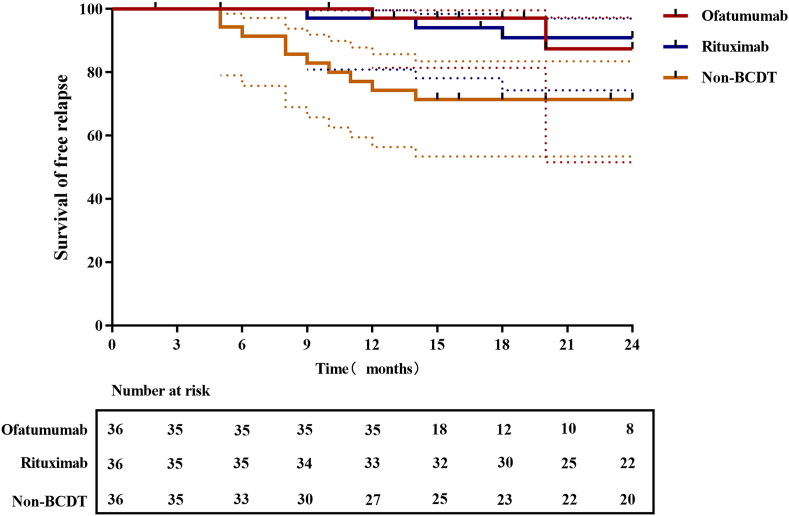
**Kaplan-Meier plot of time to relapse of the three groups** Kaplan-Meier estimates (95 ​% CI) of patients with relapse-free survival. Error bars (dashed line) indicate the 95 ​% confidence intervals.

In this study, a total of 16 relapse events were recorded from baseline time points to the end of follow-up (OFA: n ​= ​2, 5.6 ​%; RTX: n ​= ​4, 11.1 ​%; non-BCDT: n ​= ​10, 27.8 ​%; p ​< ​0.001]. One patient treated with RTX relapsed at 30 months from baseline time point. The median time to relapse was 12 months (OFA group), 16 months (RTX group), and 9 months (non-BCDT group) (p ​= ​0.019). There were no significant difference between the groups regarding the mRS score and CASE scores during relapse. Kaplan-Meier estimates showed significantly higher relapse-free survival for patients receiving OFA and RTX compared to non-BCDT (OFA vs. non-BCDT: HR, 0.193; 95 ​% CI, 0.062–0.6; p ​= ​0.007; RTX vs, non-BCDT: HR, 0.265; 95 ​% CI, 0.089–0.787; p ​= ​0.029; Fig. 5).

### Adverse events (AEs)

Safety outcomes were assessed in all patients. AEs in all groups are summarized in Table 2. Most of the adverse events were primarily mild to moderate (grade 1–3). The two most common infusion/injections-related reactions (IRR_S_) were influenza-like symptoms (12 ​%) and arrhythmia (6.5 ​%). Proportions of patients treated with RTX had more frequent arrhythmia as compared with the OFA and non-BCDT treatment groups. The commonly reported BCDT-related infections were respiratory infections of pneumocystis jiroveci and cytomegalovirus leading to BCDT withdrawal for three patients (OFA: n ​= ​2; RTX: n ​= ​1). At the 1 month follow-up, serum IgG levels were below the lower limit of normal in 24 (33.3 ​%) of the patients, IgA levels were low in 12 (16.7 ​%), and IgM levels were low in 11 (15.3 ​%). At 12 months follow-up, serum IgG levels were below the lower limit of normal in 14 (19.7 ​%) of the patients, IgA levels were low in 15 (21.1 ​%), and IgM levels were low in 10 (14.1 ​%).

### B cell depletion kinetics and reconstitution

Following RTX administration (either four weekly 100-mg infusions or a single 375 ​mg/m^2^ dose), CD19^+^ B cell depletion was achieved in 32 patients (88.9 ​%) after the initial infusion. Reconstitution of CD19^+^ B cell occurred in 26 patients (72.2 ​%) at a median of 9 months (IQR 6.25–11) from baseline. Exceptionally prolonged B cell depletion (>23 months) was observed in three patients following RTX induction. Following OFA, 30 (83.3 ​%) of patients achieved CD19^+^ B cell completely depleted since first infusion and surpassed the lower limit of normal in over 75 ​% of patients (n ​= ​6) 4–6 months after the final injection.

## Discussion

Clinical evidence is crucial for treatment decisions and consensus. Due to the rarity of NMDARE limits the ability to conduct RCTs, previous reports of benefit from BCDT have been restricted to case reports, case series, and clinical experience [[9], [10], [11], [12], [13], [14], [15]]. Therefore, we present the first comparison for the efficacy of a human anti-CD20 monoclonal antibody (OFA) to the chimeric analog (RTX) and non-BCDT in the treatment of refractory, severe NMDARE, affording insights into the diverse treatment response to different strategies. Furthermore, we demonstrate persistent cognitive dysfunction and psychosocial difficulties in severe NMDARE, highlighting the need for comprehensive rehabilitation strategies to promote further recovery.

Despite sharing a common mechanism of action targeting CD20, OFA demonstrated distinct clinical advantages in our prospective cohort. Specifically, OFA was associated with more rapid alleviation of early disease severity—as measured by CASE scores at 1 month—and a superior ability to sustain corticosteroid-free remission compared to both RTX and conventional non-BCDT regimens. This earlier treatment response aligns with emerging real-world evidence. A recent prospective cohort study similarly reported that OFA led to faster improvements in mRS and CASE scores among patients refractory to first-line immunotherapy [10]. Consistent with this, several retrospective analyses have documented marked clinical improvement within 1–3 weeks after OFA initiation, contrasting with the 6–8 week response delay often observed with RTX [14,15,23,24]. The differential efficacy between these CD20-targeting agents may be rooted in their distinct pharmacological profiles. OFA binds a unique epitope spanning both small and large extracellular loops of the CD20 receptor, resulting in stronger membrane affinity and slower dissociation kinetics. Furthermore, OFA exhibits enhanced antibody-dependent cellular cytotoxicity (ADCC) and complement-dependent cytotoxicity (CDCC) compared to RTX [17]. Additionally, pathological considerations might further under scores the use of OFA as the presence of NR1-IgG–producing B cells in cervical lymph nodes supports the rationale for deeper lymphoid tissue depletion [25], which may be better achieved by OFA owing to its subcutaneous administration. This route facilitates direct lymphatic transit prior to systemic circulation, potentially enabling more profound depletion of immune cells within lymph nodes [26]. Nevertheless, we acknowledge that clinical improvement in AE typically lags behind rapid B-cell depletion. Thus, the early differential response observed at 1 month likely represents an initial but critical divergence in clinical trajectory—an early moderation of disease severity—resulting from the combined effects of first-line immunotherapy and the early phase of B-cell depleting therapy.

In our primary analysis, BCDT was associated with a higher frequency of mRS scores ≤2 compared to non-BCDT at 3 months. Post-hoc analysis revealed no significant differences between the two BCDT groups. Univariate analysis showed that initiating BCDT earlier (within 14 days of completing first-line immunotherapy) was associated with a 4.23-fold increased odds of good outcome compared to later initiation. This findings was similar to the most comprehensive meta-analysis to date focusing on immunotherapy in NMDARE [3]. Evidence indicates that B cell subpopulation bearing the CD19^int^CD27^high^CD38^high^CD180^-^ phenotype is responsible for the selective production of antibodies in early immune responses [27]. Abnormally activated B lymphocytes stimulated by persistent antigen, may escape immunologic surveillance and differentiate into long-lived plasma cells producing antibodies. Early application with RTX may limit the buildup of the antibody-producing plasma cell pool by targeting precursor B lymphocyte subsets. It may also promote the emergence of a functional transitional regulatory B cell (Breg) pool upon B cell reconstitution, potentially contributing to long-term efficacy [28].

An important observation from our study is that while BCDT groups demonstrated superior rates of good outcome at 3 months and complete remission at 12 months, these differences were no longer significant at the final follow-up based on longitudinal disease outcomes. This implies that the principal advantage of early, potent BCDT in severe NMDARE may lie in the rapidity of neurological recovery rather than a fundamental change in the long-term outcome for all patients. A faster treatment response confers critical immediate benefits, including shorter ICU and hospital stays, a lower risk of serious complications (e.g., infections, thrombosis) from prolonged immobility, and decreased overall healthcare costs. This finding is consistent with previous literature. The early use of high-efficacy BCDT has consistently been associated with beneficial outcomes in AE [9,10,12]. A meta-analysis of 277 patients with AE documented good outcomes in 71.8 ​% of patients following RTX therapy at the last follow-up [12]. Real-world evidence from the GENERATE registry showed that 94 ​% of patients with NMDARE treated with RTX reached a favorable functional outcome [9]. More recently, the OFF-AE study reported that 91.4 ​% of patients in the OFA group achieved a favorable functional outcome [10]. Furthermore, the issue of long-term sequelae underscores the importance of rapid intervention. Despite substantial clinical remission, our cohort and others indicate that a minority of patients experience persistent cognitive deficits and residual psychosocial difficulties [28]. Large-scale analyses suggest that cognitive recovery may continue for 36 months, while self-reported deficits in emotional and social functioning often persist, restricting patients' ability to resume school or work [29]. Such sequelae have been linked to regional reductions in NMDAR density and progressive cortical alterations [30,31]. Recognizing these alterations, which may be modifiable, highlights the need for aggressive early therapy. Achieving remission more quickly could theoretically help mitigate the extent of long-term neuronal damage and improve the potential for neuroplasticity and functional rehabilitation.

In the present study, we observed relapses in 5.6 ​% of patients after OFA initiation, comparable to the OFF-AE registry study [10]. BCDT administration was significantly associated with a monophasic disease course, with the OFA and RTX groups showing 5.17-fold and 3.78-old reduced odds of relapse, respectively, compared to the non-BCDT group. This observation is consistent with a recent retrospective study as they demonstrated a substantial benefit of continued RTX infusions for reducing relapse rates in AE, especially for the adult population [32].

In this study, both OFA and RTX effectively depleted circulating B cells in 83 ​%–89 ​% of patients after the first infusion. Similar results were reported in two clinical trials involving patients with relapsing remitting multiple sclerosis (RRMS) that RTX led to a >95 ​% reduction of CD19^+^ peripheral B cells within two weeks after the last infusion [33,34]. In terms of OFA, B cell depletion was dose-dependent in the MIRROR trial [35]. By week 2, 82–85 ​% of patients had near-complete depletion of B cells [16]. We evaluated the time to reconstitution of peripheral CD19^+^ B cells in our cohort. Kinetics of B cell reconstitution differs substantially depend on the concentration of the CD20 antibodies in the lymphatic systems and route of administration. Based on data from RRMS, B lymphocyte reconstitution begins with immature transitional B cells, followed by mature naïve B cells, normalizing at 12 months post last RTX infusion, whereas memory B cells remain below lower limit of normal (LLN) for up 16 months [36]. In a pharmacokinetics (PK)–B cell modeling studies, the median B cell count returned to the LLN within 6 months (approximately 23 weeks) after OFA dosing was interrupted at 2 years [37].

Besides treatment efficacy, adverse events were assessed by the investigator at each study site. IRRs generally occurred within 24 ​h of the first injection/infusion and were mild-to-moderate. Murine-chimeric monoclona antibodies (RTX) are more likely to cause immunogenic reactions than fully human monoclona antibodies (OFA). In our cohort, infection incidence was 13.9 ​% (OFA) vs 19.4 ​% (RTX), lower than rates typically reported in RRMS trials [16]. The lower infection rate with OFA may be explained by its potent depletion of B cells and CD20^+^ T cells while apparently sparing marginal zone (MZ) B cells in the spleen and lymph nodes [26]. Although plasma cells lack CD20 expression and are unaffected by BCDT, hypogammaglobulinemia occurred in 27.8 ​% of OFA-treated patients versus 33.3 ​% of RTX-treated patients in our cohort. In RRMS patients who received rituximab, the levels of Igs were low in 7.9 ​%–22.4 ​% of patients [33]. In phase III ASCLEPIOS trial, a baseline quartile analysis from the extension study demonstrated that while the mean IgM levels decreased over time, they were maintained above the LLN from baseline to week 68, while the mean IgG levels remained stable over time with monthly ofatumumab treatment over 3.5 years compared with baseline values [38]. Of note, in severe NMDARE patients with high disease severity requiring ICU care, comorbidities and infectious complications are common. Monitoring IgG levels before and after treatment is essential to minimize serious infection risk.

Our study has several limitations. First, non-randomized treatment allocation. We were unable to mask the intervention to participants and rater. However, we followed a rigorous protocol to minimize bias due to the use of cointerventions potentially affecting outcomes. Future blind endpoint study of different anti-CD20 antibodies incorporating ofatumumab, rituximab and ocrelizumab will be crucial to fully delineate the comparative effectiveness and safety profiles of in neuroinflammatory disorders. Second, the follow-up durations differed, particularly being shorter in the ofatumumab-treated cohort, and the potential unknown confounding factors may influencing the relapse and safety outcomes. Third, detailed cognitive domains were not evaluated, standardized cognitive tests should be adopted to improve the sensitivity of long-term outcome assessments. Although clinical benefits were sustained, further investigation is needed regarding response biomarkers and the molecular mechanisms underlying relapse to these biologics during post-treatment follow-up. Additionally, peripheral blood B cell depletion may not reflect depletion within tissues. Sites like meningeal ectopic lymphoid follicles, secondary lymphoid organs, and bone marrow host intense autoimmune responses involving germinal center (GC) expansion and generation of mutated, high-affinity antibody-secreting plasma cells, central to disease progression and relapse [[39], [40], [41]]. Unraveling cellular mechanisms and conducting randomized controlled trials to assess the efficacy and safety of different BCDT protocols in neurologic disorders are needed.

Taken together, these results encourage physicians to consider both CD20 antibodies in early escalation strategies, complementing existing standard therapies. BCDT represents a significant therapeutic option in the management of severe NMDARE, offering targeted suppression of disease activity and improvement in clinical outcomes. The subcutaneous administration of ofatumumab may offer practical advantages in the long-term management of remission, potentially reducing the need for recurrent hospital visits for intravenous infusions. Monotherapy maintenance during remission can mitigate severe corticosteroid-associated side effects (e.g., osteonecrosis of the femoral head, gastrointestinal bleeding, endocrine disorders) that significantly impact psychological well-being and quality of life. Cognitive deficits resulting from long-term sequelae in NMDARE highlight the need for rapid diagnosis and adequate immunotherapy to prevent persistent damage to brain.

## Author contribution

**Data acquisition:** Baojie Wang, Yuxiu Xiao, Yufeng Chu, Chunjuan Wang, Hao Sun, Teng Huang, Danqing Qin, Xuetao Cao, Shuai Guo, Haotian Zhao, Xiumin Zhao.

**Data analysis/interpretation:** Baojie Wang, Shougang Guo.

**Statistical analysis:** Baojie Wang.

**Manuscript preparation:** Baojie Wang.

**Manuscript definition of intellectual content:** Baojie Wang, Shougang Guo.

**Manuscript editing:** Baojie Wang.

**Manuscript revision/review:** Baojie Wang, Shougang Guo.

**Manuscript final version approval:** Baojie Wang, Yuxiu Xiao, Yufeng Chu, Chunjuan Wang, Hao Sun, Teng Huang, Danqing Qin, Xuetao Cao, Shuai Guo, Haotian Zhao, Xiumin Zhao.

## Disclosure

The authors report no disclosures relevant to the manuscript.

## Study funding

This work was supported by the 10.13039/501100001809National Natural Science Foundation of China (Grant No. NSFC82371351).

## Declaration of competing interest

The authors declare that they have no known competing financial interests or personal relationships that could have appeared to influence the work reported in this paper.
