# Supplementary material for: Effectiveness of Anti-CD20 B cells depleting therapy versus conventional treatment in severe Anti-N-methyl-d-aspartate receptor encephalitis: A real-world multi-center prospective cohort study
Source: Neurotherapeutics. 2025 Oct 15;22(6):e00766. doi: 10.1016/j.neurot.2025.e00766 (PMC12664554; doi:10.1016/j.neurot.2025.e00766)
Supplement: Multimedia component 1 [file mmc1.doc]

**Effectiveness of Anti-CD20 B Cells Depleting Therapy versus Conventional treatment in Severe Anti-NMDA Receptor Encephalitis: A Real-World Multi-center Prospective Cohort Study**

**Table e-1 Baseline characteristics of rituximab-treated patients**

| **Characteristics** | **S-RTX (n=20)** | **LD-RTX (n=16)** | **P value** |
| --- | --- | --- | --- |
|
| Age, year, median (IQR) | 18 (15-38) | 31 (16-39) | 0.759 |
| Female, n (%) | 13 (65) | 9 (61.1) | 0.661 |
| Symptoms, n (%) |  |  |  |
| Seizures | 15 (75) | 11 (68.8) | 0.431 |
| Psychosis | 12 (60) | 8 (50) | 0.201 |
| Cognitive disorder | 9 (45) | 9 (56.3) | 0.157 |
| Disturbance of consciousness | 15 (75) | 11 (68.8) | 0.431 |
| Speech disturbance | 7 (35) | 7 (43.8) | 0.247 |
| Dyskinesias and movement disorders | 7 (35) | 5 (31.3) | 0.652 |
| Central hypoventilation | 2 (10) | 1 (6.3) | 0.435 |
| ICU admission, n (%) | 9 (45) | 7 (43.8) | ＞0.99 |
| ICU stay, days, median (IQR) | 21 (19-27) | 18 (16-23) | 0.237 |
| Length of hospital stay, days, median (IQR) | 34 (24-56) | 38 (25-57) | 0.598 |
| Teratoma, n (%) | 1 (5) | 1 (6.3) | ＞0.99 |
| Auxiliary examination, n (%) |  |  |  |
| MRI abnormalities | 11 (55) | 9 (56.3) | ＞0.99 |
| EEG abnormalities | 10 (50) | 10 (62.5) | 0.087 |
| CSF abnormalities | 5 (25) | 12 (75) | **<0.001** |
| CD19^+^B cell count, %, median (IQR) | 20.91 (12.58-27.78) | 24.94（17.55-27.27） | 0.334 |
| NEOS scores | 1 (1-3) | 2 (1-3) | 0.28 |
| First-line treatment, n (%) |  |  |  |
| IVIG | 19 (95) | 14 (87.5) | 0.126 |
| IVMP | 20 (100) | 16 (100) | ＞0.99 |
| PLEX | 2 (10) | 1 (6.3) | 0.435 |
| mRS score at baseline, median (IQR) | 4 (4-5) | 4 (4-5) | ＞0.99 |
| CASE score at baseline, median (IQR) | 16 (12-21) | 16 (13-19) | 0.931 |
| Cortisone at baseline, mg, mean (SD) | 107.5 (58.37) | 143 (72.34) | 0.121 |
| Time from the end of first-line treatment to B cell depleting therapy, days, median (IQR) | 12 (8-17) | 14 (10-16) | 0.36 |

**Abbreviations:** S-RTX, single dose of 375 mg/m^2^ Rituximab; LD-RTX,100mg rituximab weekly for 4 weeks; ICU, intensive care unit; MRI, magnetic resonance imaging; CSF, cerebrospinal fluid; EEG, electroencephalogram; NEOS, the NMDAR Encephalitis One-Year Functional Status; IVIG, intravenous immunoglobulin; IVMP, intravenous methylprednisolone; PLEX, plasma exchange; mRS, modified Rankin scale; CASE, Clinical Assessment Scale in Autoimmune Encephalitis; IQR, interquartile range

**Table e-2 Descriptive Data on Immunotherapy at disease onset**

| Immunotherapy | Ofatumumab  (n = 36) | Rituximab  (n = 36) | Non-BCDT  (n = 36) | *p* Value |
| --- | --- | --- | --- | --- |
| First-line immunotherapy; n (%) |  |  |  |  |
| IVMP | 36 (100) | 36(100) | 36 (100) | ＞0.99 |
| IVMP+IVIG | 32 (88.9) | 33 (91.6) | 35 (97.2) | 0.09 |
| IVMP+IVIG+PE | 4 (11.1) | 3 (8.3) | 6 (16.7) | 0.137 |
| Repeated IVIG | 1 (2.8) | 1(2.8) | 10 (27.8) | ＜0.001 |

Abbreviations: IVIG, intravenous immunoglobulin; IVMP, intravenous methylprednisolone; PLEX, plasma exchange;

**Table e-3 Long-term treatment outcome**

|  | **Ofatumumab (n=36)** | | | **Rituxumab (n=36)** | | | **Non-BCDT (n=36)** | | |
| --- | --- | --- | --- | --- | --- | --- | --- | --- | --- |
|  | mRS | CASE | mRS | | CASE | mRS | | CASE |  |
| Baseline | 4.36 (0.64) | 16.25(4.84) | 4.36 (0.72) | | 16.14 (4.16) | 4.17 (0.38) | | 14.75 (3.64) |  |
| 1 months | 2.67 (0.89) | 5.89 (3.68) | 2.78 (0.99) | | 7.91 (3.34) | 3.28 (0.66) | | 9.97 (2.42) |  |
| 3 months | 2.17 (0.91) | 3.74 (2.16) | 2.39 (1.23) | | 4.54 (2.43) | 2.72 (0.74) | | 5.83 (1.76) |  |
| 6 months | 2 (0.93) | 2.74 (1.79) | 2.22 (1.17) | | 2.77 (1.42) | 2.44 (0.88) | | 3.46 (1.22) |  |
| 9 months | 1.64 (0.99) | 1.8 (1.55) | 1.86 (1.27) | | 1.88 (1.12) | 1.86 (1.15) | | 1.89 (0.90) |  |
| 12 months | 0.47 (0.99) | 0.91 (1.17) | 0.61 (1.48) | | 1.29 (0.94) | 1.44 (1.18) | | 1 (0.80) |  |

Abbreviations：BCDT, B cell depleting therapy; mRS, modified Rankin scale; CASE, Clinical Assessment Scale in Autoimmune Encephalitis; Data are presented as mean (SD).

**Table e-4 Changes of mRS scores over time relative to baseline**

| Tukey's multiple comparisons test | | Mean Diff. | 95.00% CI of diff. | Adjusted P Value |
| --- | --- | --- | --- | --- |
| Baseline vs. 1 month | Ofatumumab | 1.69 | 1.08 to 2.31 | <0.0001 |
|  | Rituximab | 1.58 | 0.71 to 2.45 | <0.0001 |
|  | Non-BCDT | 0.89 | 0.34to 1.43 | <0.0001 |
| Baseline vs. 3 months | Ofatumumab | 2.19 | 1.58 to 2.81 | <0.0001 |
|  | Rituximab | 1.97 | 1.10to 2.84 | <0.0001 |
|  | Non-BCDT | 1.44 | 0.90 to 1.99 | <0.0001 |
| Baseline vs. 6 months | Ofatumumab | 2.36 | 1.75 to 2.97 | <0.0001 |
|  | Rituximab | 2.14 | 1.27 to 3.01 | <0.0001 |
|  | Non-BCDT | 1.72 | 1.18 to 2.27 | <0.0001 |
| Baseline vs. 9 months | Ofatumumab | 2.72 | 2.11 to 3.33 | <0.0001 |
|  | Rituximab | 2.5 | 1.63 to 3.37 | <0.0001 |
|  | Non-BCDT | 2.31 | 1.76 to 2.85 | <0.0001 |
| Baseline vs. 12 months | Ofatumumab | 3.89 | 3.28 to 4.5 | <0.0001 |
|  | Rituximab | 3.75 | 2.88to 4.62 | <0.0001 |
|  | Non-BCDT | 3.17 | 2.62 to 3.72 | <0.0001 |

Abbreviations: BCDT, B cell depleting therapy; mRS, modified Rankin scale; CIs, confidence intervals; Data are presented as mean along with 95% (CIs)

**Table e-4 Changes of CASE scores over time relative to baseline**

| Tukey's multiple comparisons test | | Mean Diff. | 95.00% CI of diff. | Adjusted P Value |
| --- | --- | --- | --- | --- |
| Baseline vs. 1 month | Ofatumumab | 10.36 | 8.40 to 12.32 | <0.0001 |
|  | Rituximab | 8.23 | 6.48 to 9.97 | <0.0001 |
|  | Non-BCDT | 5.18 | 3.74 to 6.62 | <0.0001 |
| Baseline vs. 3 months | Ofatumumab | 12.51 | 10.55 to 14.46 | <0.0001 |
|  | Rituximab | 11.60 | 9.85 to 13.34 | <0.0001 |
|  | Non-BCDT | 9.15 | 7.71 to 10.59 | <0.0001 |
| Baseline vs. 6 months | Ofatumumab | 13.51 | 11.55 to 15.46 | <0.0001 |
|  | Rituximab | 13.37 | 11.61 to 15.13 | <0.0001 |
|  | Non-BCDT | 11.29 | 9.86 to 12.73 | <0.0001 |
| Baseline vs. 9 months | Ofatumumab | 14.45 | 12.5 to 16.4 | <0.0001 |
|  | Rituximab | 14.26 | 12.5 to 16.02 | <0.0001 |
|  | Non-BCDT | 12.86 | 11.43 to 14.3 | <0.0001 |
| Baseline vs. 12 months | Ofatumumab | 15.34 | 13.38 to 17.29 | <0.0001 |
|  | Rituximab | 14.84 | 13.09 to 16.6 | <0.0001 |
|  | Non-BCDT | 13.75 | 12.31 to 15.19 | <0.0001 |

**Table e-6 36-item Short Form Health Survey (SF-36) scores**

|  | NMDAR-AE | Healthy Control | P value |
| --- | --- | --- | --- |
| PCS | 55 (46-61) | 59 (54-60) | 0.254 |
| GH | 72 (52-80) | 82 (72-87) | **0.001** |
| PF | 95 (85-100) | 100 (95-100) | **0.013** |
| RP | 100 (50-100) | 100 (75-100) | **<0.001** |
| BP | 100 (84-100) | 100 (74-100) | 0.719 |
| MCS | 51 (39-56) | 58 (43-61) | **<0.001** |
| RE | 67 (67-100) | 100 (67-100) | **0.045** |
| SF | 75 (50-75) | 88 (75-100) | **<0.001** |
| VT | 80 (60-90) | 90 (60-90) | 0.173 |
| MH | 80 (60-92) | 92 (68-92) | 0.058 |

Abbreviations: NMDAR, N-methyl-D-aspartate receptor; AE, autoimmune encephalitis; PCS, Physicalmental Component Summary; MCS, Mental Component Summary; GH, General Health; PF, Physical Functioning; RP, Role-Physical; BP, Bodily Pain;RE, Role-Emotional; VT, Vitality; SF,Social Functioning; MH, Mental Health
